# The Role of Artificial Intelligence in the Diagnosis and Management of Rheumatoid Arthritis

**DOI:** 10.3390/medicina61040689

**Published:** 2025-04-09

**Authors:** Adriana Liliana Vlad, Corina Popazu, Alina-Maria Lescai, Doina Carina Voinescu, Alexia Anastasia Ștefania Baltă

**Affiliations:** 1 Faculty of Medicine and Pharmacy, “Dunărea de Jos” University of Galați, 800008 Galați, Romania; adriana.vlad.mg3.4@gmail.com (A.L.V.); corinapopazu@yahoo.com (C.P.); carinavoinescu@gmail.com (D.C.V.); alexiabalta@yahoo.ro (A.A.Ș.B.); 2 “St. Apostle Andrei” Clinical Emergency County Hospital, 800578 Galați, Romania

**Keywords:** artificial intelligence rheumatoid arthritis, rheumatoid arthritis, management of rheumatoid arthritis, digital technologies rheumatoid arthritis, rheumatoid arthritis management, diagnosis

## Abstract

*Background and Objectives:* Artificial intelligence has emerged as a transformative tool in healthcare, offering capabilities such as early diagnosis, personalised treatment, and real-time patient monitoring. In the context of rheumatoid arthritis, a chronic autoimmune disease that demands timely intervention, artificial intelligence shows promise in overcoming diagnostic delays and optimising disease management. This study examines the role of artificial intelligence in the diagnosis and management of rheumatoid arthritis, focusing on perceived benefits, challenges, and acceptance levels among healthcare professionals and patients. *Materials and Methods:* A cross-sectional study was conducted using a detailed questionnaire distributed to 205 participants, including rheumatologists, general practitioners, and rheumatoid arthritis patients from Romania. The study used descriptive statistics, chi-square tests, and logistic regression to analyse AI acceptance in rheumatology. Data visualisation and multiple imputations addressed missing values, ensuring accuracy. Statistical significance was set at *p* < 0.05 for hypothesis testing. *Results:* Respondents with prior experience in artificial intelligence perceived it as more useful for early diagnosis and personalised management of RA (*p* < 0.001). Familiarity with artificial intelligence concepts positively correlated with acceptance in routine rheumatology practice (ρ = 1.066, *p* < 0.001). The main barriers identified were high costs (36%), lack of medical staff training (37%), and concerns regarding diagnostic accuracy (21%). Although less frequently mentioned, data privacy concerns remained relevant for a subset of respondents. The study revealed that artificial intelligence could improve diagnostic accuracy and rheumatoid arthritis monitoring, being perceived as a valuable tool by professionals familiar with digital technologies. However, 42% of participants cited the lack of data standardisation across medical systems as a major barrier, underscoring the need for effective interoperability solutions. *Conclusions:* Artificial intelligence has the potential to revolutionise rheumatoid arthritis management through faster and more accurate diagnoses, personalised treatments, and optimised monitoring. Nevertheless, challenges such as costs, staff training, and data privacy need to be addressed to ensure efficient integration into clinical practice. Educational programmes and interdisciplinary collaboration are essential to increase artificial intelligence adoption in rheumatology.

## 1. Introduction

Artificial intelligence, a field of computer science, develops systems capable of performing tasks traditionally associated with human intelligence, such as learning, reasoning, language processing, and image recognition. Using advanced algorithms, including machine learning and deep learning, artificial intelligence can analyse large datasets and make informed decisions, offering innovative solutions across various domains [1].

In healthcare, these capabilities have become particularly valuable, as artificial intelligence systems can process complex medical data, identify patterns, and assist in clinical decision-making. Machine learning, which enables computers to improve their performance based on data without explicit programming, and deep learning, which uses neural networks to interpret intricate data such as medical images, have already shown significant promise in enhancing diagnostics and patient care [2,3].

Rheumatoid arthritis diagnosis presents several challenges despite advancements in clinical practice. The disease’s heterogeneous presentation, especially in its early stages, often leads to diagnostic delays. Symptoms such as joint pain, stiffness, and swelling can overlap with other musculoskeletal disorders, making it difficult for clinicians to distinguish rheumatoid arthritis from similar conditions. Current diagnostic approaches rely on a combination of clinical symptoms, serological tests (such as rheumatoid factor and anti-cyclic citrullinated peptide antibodies), and imaging techniques like X-rays, ultrasounds, and MRIs [4]. However, these methods have limitations. Serological tests may yield false negatives in early rheumatoid arthritis, and imaging findings may not always reflect disease activity accurately. Additionally, the variability in clinical expertise and the subjective interpretation of imaging results contribute to inconsistent diagnoses.

Another limitation is the time-consuming nature of current diagnostic protocols, which often require multiple consultations and tests. This delay in diagnosis can lead to irreversible joint damage, reduced quality of life, and increased healthcare costs. Moreover, managing rheumatoid arthritis is an ongoing challenge, as clinicians must continuously monitor disease progression and adjust treatment plans based on patients’ responses, which adds to the complexity of care [5,6].

As artificial intelligence continues to reshape modern medicine, its potential extends beyond general diagnostic tools to more specialised fields. Rheumatology, a discipline dealing with complex autoimmune diseases like rheumatoid arthritis, stands to benefit greatly from artificial intelligence technologies. Artificial intelligence’s ability to analyse diverse datasets—from imaging and biomarkers to patient records—positions it as a transformative tool in addressing these challenges. Artificial intelligence algorithms can enhance early diagnosis by identifying subtle patterns in imaging data that may be overlooked by human observers, predict disease progression using longitudinal patient data, and assist in tailoring personalised treatment plans by integrating genetic, clinical, and lifestyle information. Artificial intelligence can also streamline the diagnostic process, reducing delays and improving accuracy, while offering real-time monitoring and data-driven adjustments to treatment plans, thus overcoming many of the limitations associated with current rheumatoid arthritis diagnostic and management approaches.

The objective of this research is to examine the perceptions, attitudes, and level of acceptance of artificial intelligence in the diagnosis and management of rheumatoid arthritis, both from the perspective of healthcare professionals and patients. The study aims to highlight the potential benefits, challenges, and limitations of using artificial intelligence, contributing to the understanding of how these technologies can be integrated into rheumatology clinical practice.

### 1.1. Rheumatoid Arthritis: Current Challenges in Diagnosis and Management

Rheumatoid arthritis is a chronic autoimmune inflammatory disease that primarily affects the joints, causing pain, swelling, stiffness, and, in severe cases, joint deformities [7]. Rheumatoid arthritis can also have systemic effects on other organs and is associated with long-term disabilities if not promptly treated [8].

Rheumatoid arthritis affects approximately 0.5–1% of the global population and is characterised by chronic inflammation, joint deformities, and long-term disability [9]. Early diagnosis of rheumatoid arthritis is critical, as timely interventions can reduce disease progression and prevent irreversible damage [10]. However, diagnosing rheumatoid arthritis can be challenging, requiring the correlation of clinical symptoms, serological tests (such as rheumatoid factor and anti-CCP antibodies), and imaging results [11].

Despite advancements in clinical guidelines, diagnostic errors and delays are common, particularly in atypical cases or early stages of the disease. Additionally, rheumatoid arthritis management involves continuous monitoring of treatment response and adjusting therapy according to disease progression or the emergence of adverse reactions [7]. These challenges have driven interest in adopting artificial intelligence technologies, which can offer a more accurate and faster approach to patient evaluation.

### 1.2. Applications of Artificial Intelligence in Rheumatoid Arthritis

#### 1.2.1. Early Diagnosis of Rheumatoid Arthritis

Early diagnosis refers to the process of identifying a condition in its initial stages, before the onset of severe symptoms or complications. In rheumatoid arthritis, early diagnosis is crucial for preventing disease progression and irreversible joint damage [12].

One of the most promising areas where artificial intelligence can contribute is the early detection of rheumatoid arthritis. Machine learning algorithms are capable of analysing large clinical and imaging datasets to identify patterns that may elude traditional clinical observation [13]. Convolutional neural networks (CNNs), for example, can analyse joint X-rays and MRI images to detect signs of inflammation and bone erosion with accuracy comparable to or even exceeding that of experienced rheumatologists [14].

A study by Saleh et al. (2022) demonstrated that an artificial intelligence-based model achieved a sensitivity of 94% and a specificity of 91% in diagnosing rheumatoid arthritis through the analysis of digital X-rays, using a dataset of over 10,000 images [15]. Other research highlights that machine learning algorithms can integrate clinical variables such as patient-reported symptoms, serological markers, and inflammatory biomarkers to predict the risk of developing rheumatoid arthritis in patients with undifferentiated arthritis [16,17].

#### 1.2.2. Personalised Management of Rheumatoid Arthritis

Medical monitoring involves tracking the progression of a disease over time using clinical, imaging, or biological indicators. In rheumatoid arthritis, monitoring is essential for adjusting treatments and preventing complications [18].

Continuous monitoring of rheumatoid arthritis patients is vital to ensure that treatments are tailored to individual needs and to prevent complications. Artificial intelligence -powered digital platforms, including mobile applications and wearable devices, enable real-time collection of data on patients’ symptoms, physical activity levels, and inflammatory markers [19].

For instance, wearable devices equipped with sensors can measure joint movements and monitor patient-reported pain levels. These data points are integrated by artificial intelligence algorithms to generate automated disease status reports, which can be shared with healthcare providers [20]. Additionally, chatbots and virtual assistants can support patients in managing daily symptoms and adhering to therapeutic regimens [21].

### 1.3. Challenges and Limitations of Artificial Intelligence in Rheumatoid Arthritis

A major challenge in implementing artificial intelligence in rheumatology is access to large, clean, and representative datasets. Many machine learning models are trained on retrospective datasets, which may contain errors or be influenced by confounding factors [22].

Furthermore, the standardisation of medical data remains a problem. Electronic health record systems differ across institutions and regions, making it difficult to integrate data into a compatible format for artificial intelligence algorithms [23].

The use of personal health data for training artificial intelligence algorithms raises concerns about privacy and security. Although data are often anonymised, there is a risk of re-identification, especially in complex datasets [24]. To address these concerns, healthcare organisations must adopt clear regulations regarding data usage and implement advanced security technologies, such as blockchain, to protect sensitive information [25].

Another obstacle is the reluctance of healthcare professionals and patients to embrace artificial intelligence technologies. Physicians may be sceptical about the accuracy of artificial intelligence-generated diagnoses, and patients may prefer human interaction over automated systems [26]. Studies indicate that artificial intelligence acceptance increases when algorithms are used as decision-support tools in collaboration with physicians, rather than as replacements [27,28].

### 1.4. Future Perspectives for Artificial Intelligence in Rheumatology

Advances in deep learning and the integration of multi-omic data (genetic, proteomic, and metabolomic) will enable the development of more precise and robust artificial intelligence models [29]. These models could identify specific subtypes of rheumatoid arthritis and predict individual treatment responses [30].

Successfully integrating artificial intelligence into rheumatology will require collaboration between clinicians, software engineers, and ethics experts [31]. Interdisciplinary teams can better tailor artificial intelligence solutions to clinical needs while adhering to ethical and privacy standards.

Another essential step is educating physicians and other healthcare professionals on using artificial intelligence technologies. Continuous training programmes can build trust in these systems and encourage their use in clinical practice [32].

The literature highlights numerous benefits of artificial intelligence in diagnosing and managing rheumatoid Arthritis, including early detection, personalised treatment, and disease progression monitoring. However, challenges related to data quality, privacy, and user acceptance remain. By addressing these limitations and investing in research and education, artificial intelligence has the potential to become a central component of rheumatology, significantly improving patient care.

Artificial intelligence is increasingly being recognised as a valuable tool in the diagnosis and management of rheumatoid arthritis. By leveraging advanced algorithms, artificial intelligence systems offer promising solutions to overcome challenges in early diagnosis, personalised treatment, and continuous patient monitoring. However, despite its potential, artificial intelligence adoption in rheumatology is not without limitations. Issues such as data quality, privacy concerns, and resistance from healthcare providers highlight the need for careful consideration when integrating artificial intelligence into clinical practice. Table 1 summarises the key advantages and limitations associated with the use of artificial intelligence in rheumatoid arthritis diagnosis and management.

The integration of artificial intelligence in rheumatoid arthritis diagnosis and management presents significant opportunities to improve patient outcomes through early detection, personalised care, and efficient monitoring. Nevertheless, challenges such as data quality, privacy, and system standardisation need to be addressed for successful implementation. Overcoming these limitations requires collaborative efforts from healthcare providers, technology developers, and policymakers to ensure that artificial intelligence technologies are reliable, secure, and accessible in clinical rheumatology practice.

## 2. Materials and Methods

### 2.1. Research Hypotheses

**H1.**  *Respondents with prior experience using artificial intelligence technologies are more likely to perceive them as useful for the early diagnosis and management of rheumatoid arthritis*.

Numerous studies highlight that prior experience with artificial intelligence-based technologies influences the perception of their usefulness in the early diagnosis and management of rheumatoid arthritis. For example, Soori et al. (2023) [1] emphasise the importance of user experience in the development and implementation of advanced robotics, noting that familiarity with artificial intelligence fosters greater trust and adoption. Similarly, Shi et al. (2024) [6] examine artificial intelligence applications in rheumatology, highlighting that healthcare professionals with prior experience are more inclined to utilise such technologies for rheumatoid arthritis management. In addition, Madrid-García et al. (2023) [16] provide a detailed analysis of artificial intelligence techniques adopted in rheumatology research, underscoring that prior experience with artificial intelligence enhances the perception of its clinical utility. Creagh et al. (2024) [20] demonstrate how digital technologies and artificial intelligence improve outcomes for rheumatoid arthritis patients, particularly among those already familiar with such tools.

**H2.**  *The level of acceptance of artificial intelligence in clinical practice is higher among healthcare professionals with greater general knowledge of artificial intelligence*.

The level of acceptance of artificial intelligence in medical practice is significantly higher among healthcare professionals with extensive knowledge of this technology. Taye (2023) [2] analyses how a deep understanding of artificial intelligence architecture and workflows contributes to its acceptance in medicine, including the diagnosis and management of rheumatoid arthritis. Likewise, Madrid-García et al. (2023) [16] highlight that previous studies emphasise the importance of artificial intelligence familiarity for its effective integration into rheumatology, demonstrating that the level of knowledge influences clinical adoption. Knitza et al. (2024) [19] investigate the current state of rheumatology in the digital era and underline that professionals with stronger technological training are more likely to adopt artificial intelligence in daily practice. In a study on medical education and training, Charow et al. (2021) [33] emphasise that educational programmes focused on artificial intelligence contribute to increasing acceptance of this technology among doctors and other specialists, highlighting the importance of continuous training. Thus, there is a consensus in the specialised literature that a higher level of knowledge about artificial intelligence is correlated with greater acceptance of its use in medical practice, particularly in complex fields such as rheumatology.

**H3.**  *Concerns regarding the lack of adequate training for medical staff are the main perceived obstacle to using artificial intelligence in the diagnosis and management of rheumatoid arthritis*.

Concerns related to data privacy and the lack of adequate training for medical staff are perceived as the main obstacles to the use of artificial intelligence in the diagnosis and management of rheumatoid arthritis. Jawad (2024) [25] highlights the challenges associated with data security and privacy in digital healthcare systems, emphasising that existing vulnerabilities limit trust and hinder the adoption of artificial intelligence in medical practice. In a broader context, Andraško et al. (2021) [34] analyse EU regulations on data protection and cybersecurity, underlining that the absence of clear and robust policies exacerbates healthcare professionals’ reluctance to use artificial intelligence. Sharma et al. (2024) [24] discuss the negative impact of insufficient training for medical staff on the adoption of artificial intelligence technologies, noting that a significant number of professionals regard the lack of training as a major barrier. A study by Alsaedi et al. (2024) [35] in Saudi Arabia highlights concerns expressed by healthcare professionals, who emphasise both fears regarding patient data privacy and the lack of adequate training as significant barriers to the use of artificial intelligence in rheumatology.

Thus, the specialised literature confirms that perceptions of data security and the need for continuous training are essential factors influencing the adoption of artificial intelligence in rheumatology practice.

### 2.2. Study Design

This research was designed as a cross-sectional study, aiming to investigate perceptions, attitudes, and the level of acceptance of artificial intelligence-based technologies in the diagnosis and management of rheumatoid arthritis. The study was conducted between 3 and 30 December 2024, using a detailed questionnaire for data collection—File S1.

The questionnaire was pretested on a group of 30 respondents, comprising both physicians and patients, to ensure clarity, relevance, and ease of completion. This pretesting group was purposefully selected to reflect the diversity of the main study population, enhancing the instrument’s validity. Among the physicians, there was representation from both specialised rheumatologists and general practitioners, ensuring that the questions were comprehensible to medical professionals with varying levels of expertise in rheumatology. The physicians included individuals from both public hospitals and private clinics, contributing to a broad perspective on clinical practice and technological adoption.

The patient group was equally diverse, including individuals diagnosed with rheumatoid arthritis at different stages of the disease, from newly diagnosed patients to those with long-term management experience. This demographic variation ensured that the questionnaire items were accessible and relevant to patients with differing levels of familiarity with medical terminology and digital technologies. Additionally, the pretesting group included respondents from both urban and rural settings, ensuring that the instrument was suitable for participants from diverse socio-economic and geographical backgrounds. Efforts were made to include patients with varying levels of digital literacy, allowing for the identification and correction of any potential barriers to understanding the questionnaire items.

Feedback from this demographically varied pretesting group led to several refinements in the questionnaire, including adjustments to the wording of technical terms to improve clarity for non-specialist respondents, reordering sections for a more logical flow, and ensuring that all items were uniformly understood across different respondent groups. This process helped eliminate ambiguities and improved the internal reliability of the questionnaire, ensuring that participants in the main study would interpret the questions consistently, thereby enhancing the overall robustness and validity of the research.

### 2.3. Participants

The selection of a sample of 205 respondents for this study was based on the necessity to obtain a broad perspective on the use of artificial intelligence in rheumatology, including both healthcare professionals and patients diagnosed with rheumatoid arthritis.

Table 2 presents an overview of the study participants, classified into two main groups: healthcare professionals and patients diagnosed with rheumatoid arthritis. The healthcare professionals include rheumatologists, general practitioners, and other medical specialists, while the patient group is further divided based on the duration of their diagnosis (recently diagnosed vs. chronic condition).

This classification ensures a diverse range of perspectives on the use of artificial intelligence in rheumatology.

Table 3 summarises the sources through which participants were recruited for the study. To ensure a balanced representation, participants were selected from professional networks (such as LinkedIn, WhatsApp, and academic groups), rheumatology clinics, and patient associations (including NGOs and support groups). This recruitment strategy aimed to capture insights from both medical professionals and patients regarding the application of artificial intelligence in rheumatology.

The participants come from various regions of Romania, with a balanced distribution between urban and rural areas to ensure the representativeness of perceptions regarding the use of artificial intelligence in rheumatology.

A smaller sample would have limited the external validity of the results, while a significantly larger sample would have been challenging to manage both logistically and financially.

The number of 205 participants was considered optimal, taking into account available resources such as time, costs, and access to participants, as well as the aim to ensure rigorous data collection by balancing statistical reliability with the feasibility of conducting the study. Moreover, standards in the specialised literature indicate that, in medical research and applied social sciences, sample sizes frequently range between 100 and 300 participants for exploratory or descriptive studies, making 205 an appropriate choice.

The power analysis conducted for this study also supports the selected sample size. Using a significance level of 0.05, a desired statistical power of 0.80, and an effect size of 0.3, calculations performed with G*Power 3.1 software indicated that a sample of approximately 200 participants is required to ensure adequate statistical power. Thus, a sample of 205 participants allows for the detection of significant differences between the groups analysed, reducing the risk of Type II errors. This sample size also ensures a balanced distribution between healthcare professionals and patients, contributing to the validity and robustness of the results obtained.

The participant inclusion strategy was designed to ensure a balanced and diverse representation, minimising potential selection bias. Participants were selected based on availability and accessibility, with additional efforts made to avoid voluntary response bias through the use of multiple recruitment channels, including professional networks, rheumatology clinics, and patient associations. Individuals with varying levels of digital literacy were included by providing technical support and guidance throughout the completion of questionnaires, either online or through assisted interviews. Recruitment from both urban and rural settings contributed to the diversity of the sample, while the anonymity of responses was guaranteed to encourage participation and honesty, thereby reducing the risk of bias due to social desirability or professional experience.

The level of digital literacy among participants varied significantly between healthcare professionals and patients with rheumatoid arthritis. Understanding these differences is essential for assessing the feasibility of integrating AI-based technologies into rheumatology. Table 4 presents the distribution of digital literacy levels among the two groups, highlighting their familiarity with technology and AI in medical contexts.

The analysis of digital literacy levels among participants reveals notable differences between healthcare professionals and patients with rheumatoid arthritis. Among healthcare professionals, 36.4% reported an advanced level of digital literacy, actively using AI in medical practice or health monitoring. In contrast, only 13.8% of patients had similar proficiency, indicating a lower familiarity with AI-driven technologies in patient groups (Table 4).

**Figure 1:**
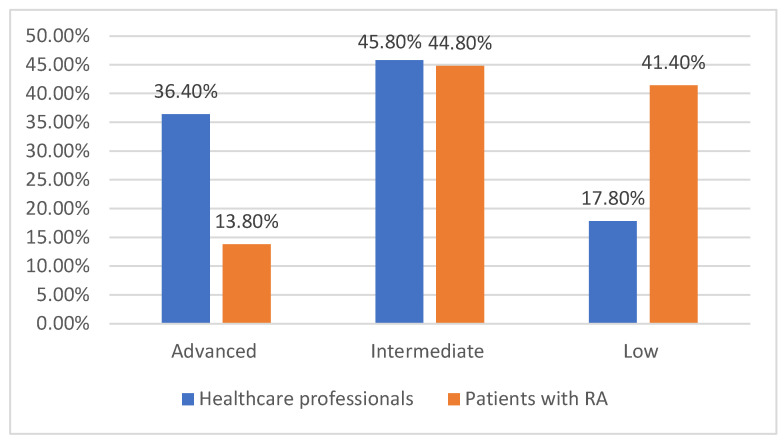
Level of digital literacy among participants.

The majority of participants fell into the intermediate category, with 45.8% of healthcare professionals and 44.8% of patients regularly using digital technology but without specific AI experience—Figure 1. This suggests that while many participants are comfortable with digital tools, additional training would be necessary to facilitate AI adoption in clinical settings and self-care.

A significant difference was observed in the low digital literacy group, where 41.4% of patients reported difficulties in using digital technology compared to only 17.8% of healthcare professionals. This disparity highlights a potential barrier to AI adoption, particularly among patients who may struggle with digital platforms.

Addressing these gaps through targeted education and the development of user-friendly AI interfaces could improve accessibility and acceptance of AI technologies in rheumatology.

The method of questionnaire completion provides insights into the digital proficiency of participants and their ability to engage with online health technologies. Table 5 compares how healthcare professionals and patients with rheumatoid arthritis completed the survey, highlighting differences in independent use and the need for assistance. These findings help identify potential barriers to AI adoption in rheumatology and the importance of user-friendly digital tools.

The method of questionnaire completion varied significantly between healthcare professionals and patients with rheumatoid arthritis, reflecting differences in digital proficiency and accessibility. Among healthcare professionals, 87.3% completed the questionnaire independently, indicating a high level of confidence in using online platforms. In contrast, only 55.2% of patients managed to complete the questionnaire without assistance, suggesting a greater need for support in navigating digital tools (Table 5).

A considerable proportion of patients, 44.8%, required assistance, compared to just 12.7% of healthcare professionals. This highlights a potential barrier to adopting AI-driven healthcare solutions, as many patients may struggle with digital interfaces. Ensuring accessibility through simplified interfaces, patient education, and support systems could enhance the successful integration of AI technologies in rheumatology.

Understanding the influence of external factors on AI acceptance is essential for assessing its integration into rheumatology. Several demographic and behavioural variables may have impacted participants’ perceptions, even though the study did not directly control for these confounding factors. Table 6 presents key associations between AI acceptance and factors such as age, gender, education level, digital literacy, and the method of questionnaire completion, highlighting statistically significant differences where applicable.

In Table 6, the analysis of confounding factors reveals significant influences on AI acceptance in rheumatology. Younger participants showed greater openness to AI, with 76% of those under 40 supporting its use, while only 51% of those over 60 were in favour, and 20% actively opposed it.

Gender differences were also observed, as 68% of men accepted AI compared to 60% of women. This slight variation, statistically significant with *p* = 0.04, suggests a more cautious approach among female participants, particularly regarding data security and diagnostic accuracy.

Education level played a crucial role, with 74% of respondents with higher education supporting AI, compared to lower acceptance among those with secondary education.

Digital literacy strongly influenced AI perception, with 84% of participants with high digital proficiency accepting AI, whereas only 42% of those with low digital literacy supported its implementation. This relationship was statistically significant, with *p* < 0.001, highlighting the importance of technological competence in AI adoption.

The method of questionnaire completion also reflected differences in AI perception. Among those who completed the survey independently, AI acceptance was notably higher, whereas participants who required assistance, representing 26.3% of the sample, were more sceptical. This difference was statistically significant, with *p* = 0.03, suggesting that digital accessibility challenges could act as a barrier to AI adoption.

### 2.4. Research Instrument

The data collection instrument was a structured questionnaire developed and administered via the Google Forms platform. The questionnaire was designed based on the research goals, objectives, and a review of relevant literature. It was organised into 10 distinct sections, each addressing a specific dimension of artificial intelligence usage in rheumatology.

The questionnaire sections covered the following: demographic data, general knowledge of artificial intelligence, perceptions of artificial intelligence’s benefits and limitations, identified challenges, prospects for AI integration in clinical practice, data confidentiality, patient acceptance of AI technologies, impact on privacy and ethics, future directions for artificial intelligence in rheumatology, and general feedback.

It included 27 closed-ended and multiple-choice questions. A Likert scale was used to measure agreement levels with various statements, providing a robust basis for statistical analysis. A Likert scale was used to measure agreement levels with various statements, offering a structured and reliable method for capturing participants’ perceptions and attitudes. This scale consisted of five points, ranging from 1 (Strongly Disagree) to 5 (Strongly Agree), allowing respondents to express varying degrees of agreement or disagreement. The intermediate points, such as 2 (Disagree), 3 (Neutral), and 4 (Agree), provided nuance and depth to the collected data, making it suitable for statistical analysis. The Likert scale’s ordinal nature facilitated the quantification of subjective opinions, enabling the application of descriptive and inferential statistical techniques to assess trends, correlations, and differences within the dataset.

### 2.5. Data Collection

The questionnaire was distributed using a mixed-strategy approach, leveraging social media platforms (Facebook, LinkedIn), email, and active professional WhatsApp groups. This approach facilitated access to a diverse audience of healthcare professionals and patients, contributing to the collection of a representative sample.

Respondents were able to complete the questionnaire online using electronic devices such as mobile phones, tablets, or computers, ensuring an accessible and efficient data collection process.

### 2.6. Informed Consent

Before completing the questionnaire, participants were informed about the purpose and voluntary nature of the study. On the first page of the questionnaire, respondents were presented with an informed consent document detailing the research objectives, their rights, data confidentiality, and the option to withdraw from the study at any time. Participation was conditional on ticking the corresponding informed consent checkbox before accessing the questions.

### 2.7. Data Analysis

The collected data were analysed using SPSS version 26, a well-established statistical software for quantitative data analysis. Both descriptive and inferential statistical techniques were applied during data processing.

### 2.8. Statistical Methods

Descriptive statistics were used to summarise the data, presenting frequencies, percentages, and means to illustrate the distribution of responses. Categorical variables, such as the level of acceptance of artificial intelligence (AI) and perceived barriers, were analysed using cross-tabulations to compare groups.

Inferential statistics included chi-square tests to assess associations between categorical variables, such as the relationship between AI experience and perception of its usefulness. A *p*-value threshold of 0.05 was applied to determine statistical significance. Additionally, logistic regression analysis was conducted to evaluate predictors of AI acceptance, controlling for demographic factors such as age, gender, and education level.

Data visualisation techniques, including bar charts and pie charts, were used to illustrate key findings. The analysis was performed using statistical software to ensure accuracy and reliability. Missing data were handled through multiple imputation techniques where necessary, ensuring the integrity of the dataset.

### 2.9. Instrument Reliability

The reliability of the questionnaire was evaluated by calculating Cronbach’s Alpha coefficient for each section and for the entire instrument. The values obtained were above the threshold of 0.7, indicating adequate internal consistency of the items within each dimension.

Sections addressing perceptions of artificial intelligence benefits, challenges, limitations, data confidentiality and ethics, as well as future artificial intelligence applications in rheumatology, achieved Cronbach’s Alpha values above 0.8, reflecting very high reliability.

To assess the stability of responses over time, a test–retest was conducted on a subset of participants after a two-week interval. The correlation coefficient obtained for each section was consistent, with most values ranging between 0.8 and 0.88, demonstrating high temporal stability of responses (Table 7). This temporal consistency suggests that participants provided similar responses to the same questions even after a time interval, supporting the instrument’s reliability over time.

The analysis presented in Table 7 confirmed that the research instrument is reliable and yields consistent results, making it suitable for investigating perceptions and attitudes related to the use of artificial intelligence in the diagnosis and management of rheumatoid arthritis.

Construct validity was examined to ensure that the questionnaire accurately measured the theoretical constructs it was intended to assess. An exploratory factor analysis (EFA) was performed using principal component analysis with varimax rotation. The Kaiser-Meyer-Olkin (KMO) measure of sampling adequacy was 0.84, and Bartlett’s test of sphericity was significant (*p* < 0.001), indicating that the data were suitable for factor analysis. The EFA extracted five distinct factors corresponding to the main dimensions of the questionnaire, each with eigenvalues greater than 1. Factor loadings above 0.6 for each item confirmed that the items were appropriately grouped under their respective constructs, providing strong evidence of construct validity.

Face validity was established by soliciting feedback from a panel of experts in rheumatology, medical research, and artificial intelligence. Experts reviewed the questionnaire to assess whether the items appeared to measure the intended concepts. Minor modifications were made based on their feedback to improve clarity, ensure comprehensiveness, and align with current artificial intelligence applications in rheumatology. Participants also provided feedback during a pilot study phase, indicating that the questionnaire was clear, well-structured, and relevant to their experiences, further supporting face validity.

Content validity was ensured through an extensive literature review on artificial intelligence applications in rheumatology and related medical fields. The questionnaire items were developed to cover all relevant domains, including artificial intelligence benefits, challenges, ethical considerations, data confidentiality, and future applications. A panel of five experts in rheumatology and medical informatics evaluated each item to determine its relevance and representativeness. The Content Validity Index (CVI) was calculated, with all items receiving a score above 0.78, indicating excellent content validity.

### 2.10. Ethical Considerations

The study was conducted in accordance with ethical principles, ensuring the confidentiality and anonymisation of participants’ data. The information collected was used exclusively for research purposes, in compliance with GDPR regulations. Respondents were informed of their rights, including the option to withdraw from the study at any time without any consequences.

This robust methodology facilitated the collection of relevant data, contributing to the identification of perceptions and challenges associated with the use of artificial intelligence in the diagnosis and management of rheumatoid arthritis. The findings provide a valuable foundation for developing future strategies to integrate artificial intelligence into clinical practice.

Addressing potential biases in artificial intelligence models and the ethical considerations associated with decision-making in healthcare are essential topics for the responsible implementation of this technology. Biases can arise from various sources, including unrepresentative training data, variable selection, and result interpretation. Medical data used to train algorithms may not reflect the diversity of the population, leading to biassed outcomes, particularly for minority or vulnerable groups. Additionally, the variables selected for modelling can amplify existing disparities, and the interpretation of results by medical professionals may introduce further bias.

To mitigate these risks, it is necessary to diversify datasets to include a broad range of demographic and socio-economic characteristics. Implementing external audits of algorithms and using explainable models can help identify and correct biases, providing transparency in artificial intelligence-driven decision-making. Moreover, continuous testing of algorithms post-implementation and involving medical professionals in assessing their performance contribute to maintaining high standards of fairness and accuracy.

Ethical considerations related to the use of artificial intelligence in healthcare include data privacy protection, accountability for medical decisions, and equitable access to these technologies. Artificial intelligence systems require large volumes of data, raising concerns about security and privacy, particularly in the absence of clear regulations. Furthermore, accountability for artificial intelligence-assisted medical decisions poses significant legal and ethical challenges. Algorithmic transparency is crucial for user trust, and patients must be informed about the use of artificial intelligence in their care processes.

Adopting clear regulations, providing continuous education for medical staff, and promoting interoperability between healthcare systems are necessary measures for the efficient and ethical use of artificial intelligence. Without these measures, there is a risk of perpetuating existing inequalities in the healthcare system and losing trust in new technologies. Therefore, addressing biases and adhering to ethical considerations are fundamental pillars for integrating artificial intelligence into modern healthcare.

## 3. Results

### 3.1. Level of Acceptance of Artificial Intelligence in Medical Practice

Table 8 presents the level of acceptance of artificial intelligence (AI) in medical practice, based on responses from healthcare professionals and patients with rheumatoid arthritis (RA). The results highlight a generally positive perception of AI, with the majority of participants recognising its potential benefits in diagnosis and treatment. However, some respondents remain neutral or sceptical, citing concerns such as costs, training gaps, and data privacy issues. The distribution of responses is detailed below.

The results from Table 8 indicate a high level of acceptance of artificial intelligence (AI) among both healthcare professionals and patients with rheumatoid arthritis (RA). Overall, 69.5% of healthcare professionals and 62.1% of patients consider AI useful for diagnosis and treatment. However, 13.2% of participants expressed reservations about AI implementation in rheumatology, citing barriers such as high costs, lack of medical staff training, and concerns regarding data confidentiality.

**Figure 2:**
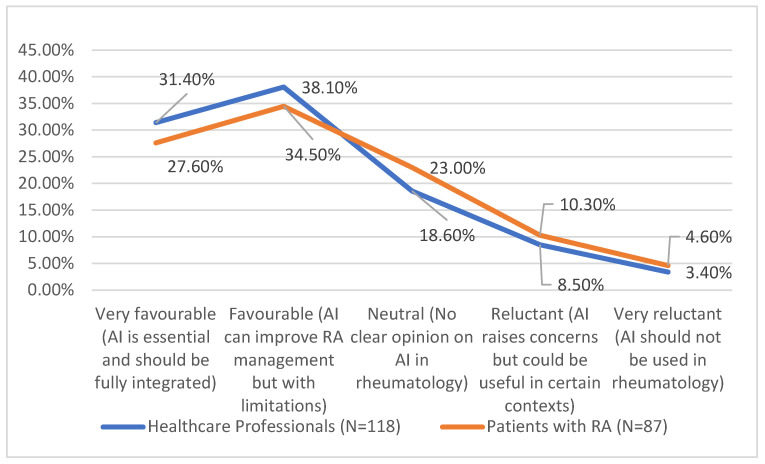
Level of acceptance of artificial intelligence in medical practice.

The results from Figure 2 show that the perceptions of AI in rheumatology are largely similar between the two groups, with only minor variations. The proportion of participants who view AI favourably is comparable, with a difference of just 3.6 percentage points, indicating a generally positive attitude towards its potential benefits. The neutral category shows a slightly larger difference of 4.4 percentage points, suggesting that one group is somewhat more undecided about AI’s role in rheumatology.

Reluctance towards AI remains relatively low in both groups, with only small differences of 1.8 percentage points for those expressing concerns but acknowledging potential usefulness and 1.2 percentage points for those who strongly oppose AI use. These small variations indicate that while some scepticism exists, the overall trends in AI acceptance remain consistent across both groups.

### 3.2. Users’ Experience with Artificial Intelligence in the Early Management of RA

Table 9 presents the relationship between prior experience with artificial intelligence and the perception of its utility in rheumatoid arthritis. The data compares individuals who have previously used AI-based technologies with those who have not, examining their attitudes towards its usefulness in clinical practice.

The results from Table 9 indicate that those with prior AI experience are more likely to consider it useful, with 84.5% expressing a positive perception. In contrast, only 57.3% of participants without prior experience view AI as beneficial. Neutral attitudes are more common among those who have not used AI before, at 23.7%, compared to 9.5% among experienced users. Similarly, reluctance towards AI is higher in those without prior experience, with 19% expressing reservations, compared to just 6% among those who have used AI.

These findings suggest that direct exposure to AI-based technologies may positively influence perceptions of their utility in managing rheumatoid arthritis.

**Figure 3:**
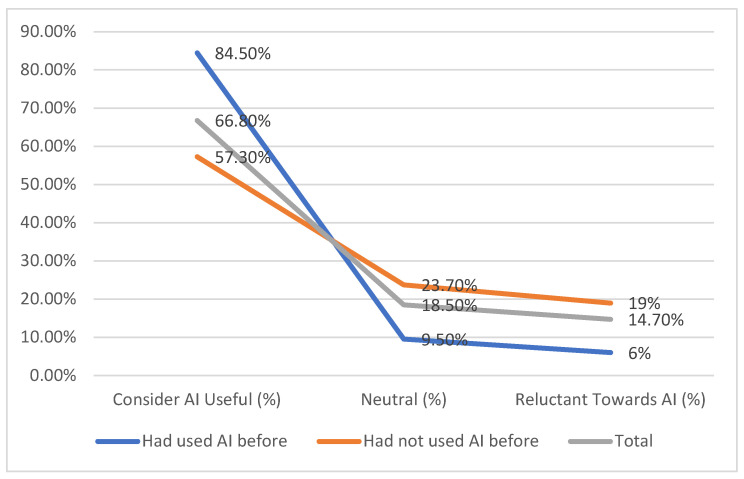
Relationship between AI experience and perception of AI utility in RA.

Although there is a noticeable gap in attitudes, the overall trend suggests that familiarity with AI is associated with a more favourable perception of its usefulness. While more than half of those without experience still found AI beneficial, their level of support was lower than that of experienced users. These findings highlight the potential impact of exposure and education in shaping opinions about AI in clinical settings (Figure 3).

### 3.3. Level of AI Acceptance in Medical Practice

Table 10 presents the relationship between prior experience with artificial intelligence and the perception of its utility in rheumatoid arthritis. The data compares individuals who have previously used AI-based technologies with those who have not, examining their attitudes towards its usefulness in clinical practice.

The results from Table 10 show that individuals with very good AI knowledge are the most supportive of its integration, with 93% expressing a positive attitude. Support decreases as AI knowledge declines, with only 42.1% of those with low or no knowledge favouring its use. Neutral opinions are more common among those with limited knowledge, reaching 33% in the lowest knowledge group compared to just 5% among those highly familiar with AI. Opposition to AI is also more pronounced in participants with little or no AI knowledge, at 24.9%, compared to only 2% among those with strong AI understanding.

These findings suggest that greater awareness and understanding of AI are associated with higher acceptance of its role in rheumatology, highlighting the potential importance of education and exposure in shaping positive attitudes.

### 3.4. Barriers to AI Adoption in Rheumatology

The study examined the most common barriers to the adoption of artificial intelligence in rheumatology, as perceived by both healthcare professionals and patients. Table 11 presents the distribution of these barriers, highlighting key concerns that may impact the successful implementation of AI in clinical practice.

In Table 11, high costs were identified as a significant barrier by both groups, with 36.1% of respondents citing this issue. Patients were slightly more concerned about costs, with 40.2% selecting this option compared to 33.1% of healthcare professionals. Lack of staff training emerged as the most frequently reported barrier overall, with 37.6% of participants highlighting it as a concern. Healthcare professionals expressed the greatest concern in this area, at 39.8%, while 33.3% of patients also saw it as a challenge. Data privacy concerns were noted by 21% of respondents, with a higher proportion among patients at 27.5% compared to 16.1% of healthcare professionals. Other barriers, such as limited insurance coverage and miscellaneous concerns, were reported less frequently.

These findings suggest that financial constraints, insufficient training, and data security concerns are key obstacles to AI adoption in rheumatology. Addressing these issues through policy changes, education, and secure data management practices could facilitate greater acceptance and integration of AI technologies.

**Figure 4:**
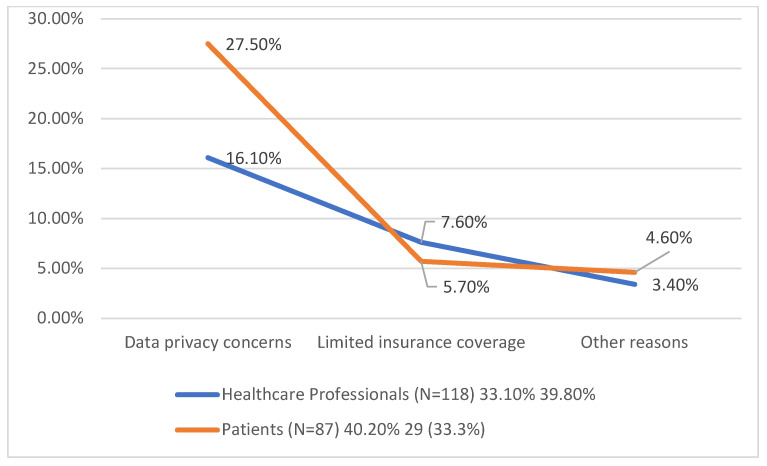
Most common barriers to AI use.

In Figure 4, while the differences between groups are not extreme, patients tend to be more concerned about financial and privacy-related issues, whereas healthcare professionals emphasise the need for adequate training. These findings suggest that addressing both financial and educational barriers could enhance AI adoption in rheumatology.

### 3.5. Differences in Attitudes Between Healthcare Professionals and Patients with Rheumatoid Arthritis

In Table 12, attitudes towards the use of artificial intelligence (AI) varied between healthcare professionals and patients with rheumatoid arthritis (RA). While professionals were more inclined to accept AI as a diagnostic and management tool, patients were more concerned about data privacy and preferred human interaction.

In Table 12, the data show both similarities and differences in how healthcare professionals and patients with rheumatoid arthritis (RA) perceive artificial intelligence (AI). While there is general agreement on its potential benefits, certain concerns vary between the two groups.

Healthcare professionals are more likely to see AI as beneficial for RA diagnosis (73%) compared to patients (62%), suggesting a stronger confidence in AI’s role in medical decision-making. However, both groups recognise AI’s potential in disease monitoring, though acceptance is higher among professionals (65%) than patients (49%).

Concerns about AI differ in emphasis. Patients are more worried about data privacy (27.5%) than professionals (19%), reflecting a greater sensitivity to personal information security. Conversely, professionals express greater concern over algorithm accuracy (35%) compared to patients (18%), indicating a focus on clinical reliability.

One area where perceptions are relatively aligned is the impact of AI on the doctor-patient relationship. While professionals (12%) and patients (18%) show some concern, the difference is not substantial, suggesting a shared understanding of the importance of human interaction in healthcare.

**Figure 5:**
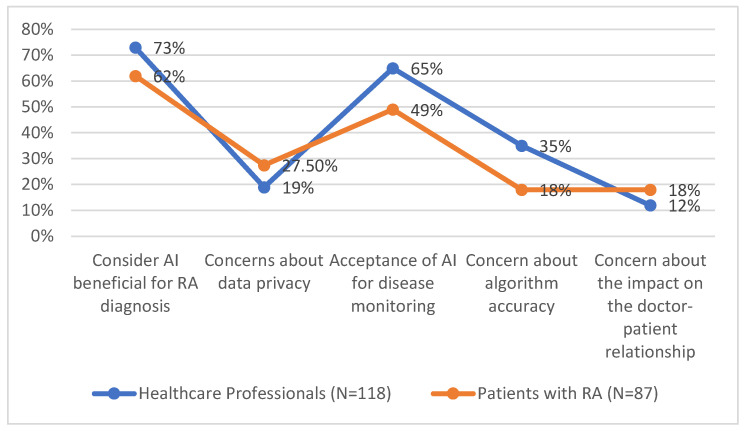
Differences in attitudes towards AI between healthcare professionals and patients with RA.

Overall, while there are areas of overlap in perspectives, the data indicate that professionals prioritise AI’s technical reliability (Figure 5), whereas patients focus more on privacy and the human aspect of medical care.

Table 13 explores how demographic variables influence the perception of AI use in rheumatology, highlighting differences in acceptance levels and key concerns across age, gender, and education levels. The data suggest that younger individuals and those with higher education are more receptive to AI, while older participants and those with lower education levels express greater scepticism. Additionally, gender differences reveal varying priorities, with men focusing on data standardisation and women being more concerned about privacy and diagnostic accuracy. The table below provides a detailed breakdown of these findings.

In Table 13, the results suggest that demographic factors influence the acceptance and concerns regarding AI use in rheumatology. Younger participants showed the highest acceptance of AI, with 76% of those under 40 supporting its use, while acceptance decreased with age. Those over 60 were the most sceptical, citing the complexity of AI and a lack of trust as primary concerns.

Gender differences were also observed, with men demonstrating a slightly higher acceptance rate (68%) compared to women (60%). Men were more concerned about the lack of data standardisation, whereas women highlighted privacy risks and potential diagnostic errors as key issues.

Education level played a significant role in shaping attitudes towards AI. Participants with higher education showed greater acceptance (74%) but were more cautious about the reliability of AI algorithms. In contrast, those with secondary education had a lower acceptance rate (58%) and expressed concerns about over-reliance on technology in medical decision-making.

Overall, the data indicate that younger, highly educated individuals tend to be more receptive to AI in rheumatology, while older participants and those with lower education levels are more sceptical, often due to concerns about complexity, trust, and the potential risks associated with AI implementation.

## 4. Discussion

Within the scope of the first hypothesis, the analysis of the results highlights statistically significant differences between participants with and without prior experience using artificial intelligence technologies. These findings align with the existing literature, which emphasises that familiarity with artificial intelligence significantly influences perceptions of its utility and adoption in healthcare.

For example, a study conducted by Pajalic et al. (2024) found that healthcare professionals who are more familiar with artificial intelligence technologies tend to perceive them as more beneficial in managing chronic diseases, including rheumatoid arthritis [36]. This can be attributed to a deeper understanding of how artificial intelligence can contribute to patient monitoring, personalised treatment, and enhanced clinical decision-making.

Moreover, the literature supports the idea that prior experience facilitates the acceptance of new technologies, as outlined by the Technology Acceptance Model (TAM). According to research by Ezhil Grace and Thandaiah Prabu (2023), perceptions of usefulness and ease of use positively influence the intention to adopt artificial intelligence, an effect that is amplified among those who have previously interacted with such technological solutions [37]. In the context of rheumatoid arthritis, artificial intelligence is used for tasks such as medical image analysis, identification of predictive markers, and optimisation of therapeutic regimens—functions that are better understood by those with prior experience.

On the other hand, a lack of experience can result in more conservative perceptions of artificial intelligence. This is supported by Palominos (2019), who observed that individuals without prior exposure to artificial intelligence often exhibit reluctance, fears of replacing the human factor, or doubts regarding the technology’s accuracy [38].

Furthermore, artificial intelligence is recognised for its potential to enhance personalised care for patients with rheumatoid arthritis. Advanced algorithms can rapidly analyse large volumes of clinical data, identifying subtle patterns that may be overlooked in human assessments. According to a study by Momtazmanesh (2022), these capabilities provide benefits for both physicians and patients, improving the quality and efficiency of care delivery [39].

The statistical analysis results from this study are supported by the existing literature, which confirms the significant role of prior experience in shaping perceptions of artificial intelligence. These findings highlight the importance of education and training in the use of advanced technologies to reduce psychological and operational barriers associated with artificial intelligence adoption in healthcare.

With regard to the second hypothesis, the analysis results—indicating a significant positive correlation between the level of understanding of the concept of artificial intelligence and perceptions of its integration into routine rheumatology practice—are consistent with conclusions from the literature. Previous studies have demonstrated that digital literacy and comprehension of emerging technologies directly influence the acceptance and adoption of these innovations across various medical fields [23,40].

For example, according to an analysis by Khan (2024), healthcare professionals with strong knowledge of artificial intelligence tend to hold more favourable attitudes towards its use, perceiving it as a valuable tool for optimising clinical processes and decision-making [41].

In rheumatology, artificial intelligence has the potential to enhance early diagnosis, personalise treatments, and monitor the progression of chronic conditions such as rheumatoid arthritis. Arefin (2024) highlights that the application of artificial intelligence techniques, including machine learning, in musculoskeletal imaging and clinical data analysis can significantly improve diagnostic accuracy and therapeutic efficiency [42]. However, the adoption of artificial intelligence technologies is often hindered by healthcare practitioners’ limited familiarity with these concepts.

Similarly, the study by Afrazeh and Shomalzadeh (2024) found that professionals with a deeper understanding of artificial intelligence’s functionality and limitations are more likely to accept its integration into daily routines, recognising its value in reducing administrative burdens and improving doctor-patient relationships [43]. Our study similarly underscores the importance of increasing knowledge about artificial intelligence to encourage its acceptance and adoption.

On the other hand, the literature also highlights potential barriers, such as the lack of specialised artificial intelligence education in traditional medical training and fears about human replacement. According to Ardichvili (2022), a higher level of understanding of artificial intelligence can mitigate these fears, shifting the perception of the technology from a threat to an opportunity for human–machine collaboration [44].

The results confirm the need for educational programmes aimed at improving artificial intelligence literacy, which could help reduce resistance to its integration into clinical practice. Promoting a high level of understanding can positively impact the acceptance and use of artificial intelligence in rheumatology, aligning with trends and conclusions reported in the literature.

Regarding Hypothesis 3, the findings of this study are consistent with trends highlighted in the literature, which emphasise the multiple challenges of implementing artificial intelligence in the medical field, particularly in the treatment of chronic diseases such as rheumatoid arthritis.

A recurring issue in the literature is the high cost of implementing artificial intelligence, frequently identified as a significant barrier. Studies have shown that adopting artificial intelligence requires substantial investments in infrastructure, equipment, and software updates, which may discourage its use, especially in healthcare systems with limited resources [45,46]. This barrier was the most commonly mentioned by participants in this study, often associated with moderate or neutral reservations about artificial intelligence adoption.

In terms of the lack of training for medical staff, the literature highlights that this significantly affects artificial intelligence adoption. Many healthcare professionals feel they lack the technical skills needed to effectively integrate artificial intelligence into clinical practice [35]. This finding is supported by the results of this study, where this factor was the second most frequently cited challenge.

Uncertainty about the accuracy of artificial intelligence-generated diagnoses has been highlighted in numerous articles as a major source of scepticism. Research by Qamar (2024) and Bi et al. (2019) shows that while artificial intelligence algorithms can enhance diagnostic accuracy, errors or false-positive/negative results can undermine physicians’ trust in these systems [47,48]. The findings of this study reflect this issue, although the proportion of respondents who view accuracy as a challenge is smaller compared to other barriers.

Patient data privacy concerns, though reported by a smaller number of respondents, are widely discussed in the literature. According to the study by Andraško et al. (2021), risks associated with security breaches and unauthorised use of personal data hinder artificial intelligence adoption, particularly in countries with strict data protection regulations (e.g., GDPR in the European Union) [34]. Although this issue was less significant among the respondents in this study, the literature underscores its long-term importance.

The literature frequently emphasises that data privacy concerns and uncertainty about the accuracy of algorithms are among the main perceived barriers to artificial intelligence adoption in medicine. According to Engelhardt (2017), fears related to the inappropriate use of sensitive data undermine the trust of both patients and healthcare professionals in artificial intelligence technologies [49]. This aligns with the distribution of responses in our analysis, where concerns are more pronounced when privacy and accuracy are identified as key challenges.

As noted by Mansour et al. (2024), high costs and the lack of adequate infrastructure for artificial intelligence implementation represent significant barriers, particularly in resource-limited healthcare systems [50]. Our findings reveal that many respondents perceive costs as a major challenge; however, they tend to be less concerned about the use of personal data. This suggests that the issue of costs is seen more as a logistical barrier rather than an ethical or privacy-related one.

The study by Charow (2021) highlights that the lack of training among healthcare professionals to integrate artificial intelligence into their practices is a major challenge [33]. The response distribution in our analysis shows that, although many respondents remain neutral regarding concerns about personal data, they do recognise the lack of training as a significant issue. This suggests that ethical concerns (related to data privacy) and operational challenges (related to training) are perceived as distinct by respondents.

The statistical significance of the association between the analysed variables is consistent with studies that emphasise the connection between individual concerns about artificial intelligence use and the perception of risks. For example, Shin et al. (2022) state that risk perception is significantly influenced by understanding how personal data are utilised in algorithms [51]. Thus, the chi-square findings from our analysis are supported by similar evidence in the literature.

Some studies, such as those by Kim and Lee (2024), suggest that perceptions of artificial intelligence are influenced by socio-cultural factors, which may explain why certain challenges, such as privacy, hold greater importance for specific groups [52]. This diversity helps explain why our respondents are divided between neutral and specific concerns.

## 5. Conclusions

The study highlights the essential role of artificial intelligence in the diagnosis and management of rheumatoid arthritis, emphasising its potential to transform rheumatology practice. Respondents perceived artificial intelligence as particularly useful for early diagnosis and treatment personalisation, but its implementation remains influenced by several factors. Familiarity with artificial intelligence technologies proved to be a significant determinant of their acceptance, with participants possessing a higher level of artificial intelligence knowledge being more likely to view it as beneficial in clinical practice. This finding underscores the need for education and training programmes for healthcare professionals.

The results indicated significant barriers to artificial intelligence adoption, with the most common being high implementation costs, lack of staff training, and concerns about data privacy. While uncertainty regarding diagnostic accuracy was mentioned, it was not considered a major obstacle for all participants. Respondents acknowledged that artificial intelligence technologies could enhance disease progression monitoring, optimise therapeutic responses, and reduce the risk of complications. However, challenges such as data standardisation across medical systems and the integration of artificial intelligence into existing infrastructures were identified as significant logistical issues.

The study confirmed a positive correlation between digital literacy levels and the degree of artificial intelligence acceptance in rheumatology. Respondents with prior experience using artificial intelligence technologies reported higher levels of trust in algorithm-assisted diagnoses and viewed artificial intelligence as a valuable support tool rather than a replacement for human expertise. At the same time, respondents stressed the importance of protecting patient data privacy and implementing clear regulations to prevent unauthorised uses.

The findings of this study underscore the need for interdisciplinary collaboration among healthcare professionals, technology developers, and policymakers to maximise the benefits of artificial intelligence. Addressing the identified challenges and promoting education in artificial intelligence can facilitate the integration of this revolutionary technology, contributing to improved patient care for rheumatoid arthritis and the optimisation of healthcare resources.

Regarding clinical practice in the healthcare facility where I work, the first step in utilising artificial intelligence is the digitalization of medical procedures, starting with the anamnesis, care plan, and case management plan. The most important aspect is access to a computerised database of the patient’s medical history.

Potential biases inherent in self-reported data may affect the accuracy of the results, as participants’ responses could be influenced by their subjective perceptions and experiences. Although the geographical and occupational diversity of the sample was an objective, it may not be fully representative of the entire population of patients and professionals in the field of rheumatology, thereby limiting the generalisability of the conclusions. Ethical considerations regarding the use of artificial intelligence in rheumatology raise important questions related to data confidentiality and the potential impact on the patient-clinician relationship, particularly as artificial intelligence technologies may influence clinical decision-making and the level of trust between parties.

Future research should explore longitudinal studies to assess the long-term impact of artificial intelligence integration in rheumatology, focusing on clinical outcomes, patient satisfaction, and cost-effectiveness. Comparative studies across different healthcare systems and geographic regions would provide insights into how contextual factors influence artificial intelligence adoption. Additionally, research on the development of user-friendly artificial intelligence tools tailored to the specific needs of rheumatology practices could enhance acceptance and usability among healthcare providers.

Studies investigating the ethical implications of artificial intelligence in rheumatology, particularly regarding data privacy, algorithmic transparency, and decision-making accountability, are essential. Further research is also needed to evaluate the effectiveness of artificial intelligence training programmes for healthcare professionals and their impact on artificial intelligence adoption rates and clinical performance.

In clinical practice, a foundational step for artificial intelligence integration is the digitalisation of medical procedures, starting with the anamnesis, care plans, and case management protocols. Establishing a centralised, computerised database of patients’ medical histories is critical for enabling artificial intelligence systems to access comprehensive data for accurate analysis and decision-making. Healthcare facilities should prioritise investments in secure digital infrastructures, staff training in artificial intelligence technologies, and collaboration with technology developers to customise artificial intelligence solutions for rheumatology. Implementing artificial intelligence-assisted diagnostic tools, treatment recommendation systems, and patient monitoring platforms can significantly enhance the quality of care, reduce administrative burdens, and optimise resource allocation. Regular evaluation and updates of artificial intelligence systems, based on clinical feedback and technological advancements, will ensure their continuous relevance and effectiveness in rheumatoid arthritis management.

## Figures and Tables

**Table 1:** Advantages and limitations associated with the use of artificial intelligence in rheumatoid arthritis diagnosis and management.

Advantages	Limitations
Artificial intelligence algorithms can analyse X-rays and MRIs to detect inflammation and bone erosion, enabling faster and more accurate diagnosis.	Incomplete or inconsistent medical data across different healthcare institutions can affect artificial intelligence accuracy.
Artificial intelligence platforms integrate clinical data, biomarkers, and patient-reported symptoms to tailor individualised treatment plans.	The use of personal health data raises concerns about privacy, data breaches, and unauthorised access.
Wearable devices track joint movements, pain levels, and inflammatory markers in real-time, with AI generating automated reports for healthcare providers.	Variability in electronic health record systems across hospitals and regions makes data integration challenging.
Artificial intelligence models, including machine learning and deep learning, achieve high sensitivity and specificity in RA detection, sometimes surpassing human experts.	Healthcare professionals may be sceptical about artificial intelligence’s reliability, and patients may prefer human interaction over automated tools.
Artificial intelligence reduces time spent on manual data analysis, allowing clinicians to focus more on patient care and decision-making.	Initial costs of artificial intelligence integration, including software, hardware, and training, can be a barrier, especially in resource-limited settings.

**Table 2:** Study participants.

**Category**	**N**	**Percentage (%)**
**Healthcare Professionals**	118	
**- Rheumatologists**	45	38.13
**- General Practitioners**	53	44.92
**- Other Medical Specialists**	20	16.95
**Patients with Rheumatoid Arthritis**	87	
**- Recently Diagnosed (<2 years)**	29	33.33
**- Chronic Condition (>2 years)**	58	66.67
**Total Participants**	205	100.0

**Table 3:** Recruitment sources.

Source	N	Percentage (%)
**Professional Networks** (LinkedIn, WhatsApp, academic groups)	72	35.1
**Rheumatology Clinics**	83	40.5
**Patient Associations** (NGOs, support groups)	50	24.4
**Total Participants**	205	100.0

**Table 4:** Level of digital literacy among participants.

Digital Literacy Level	Healthcare Professionals (N = 118)	Patients with RA (N = 87)	Total (%)
**Advanced** (uses AI in medical practice/health monitoring)	43 (36.4%)	12 (13.8%)	**55 (26.8%)**
**Intermediate** (regular technology user, but no specific AI experience)	54 (45.8%)	39 (44.8%)	**93 (45.4%)**
**Low** (difficulties in using digital technology)	21 (17.8%)	36 (41.4%)	**57 (27.8%)**

**Table 5:** Method of questionnaire completion.

Method of Completion	Healthcare Professionals (N = 118)	Patients with RA (N = 87)	Total (%)
**Independent** (online, without assistance)	103 (87.3%)	48 (55.2%)	**151 (73.7%)**
**With assistance** (explanations, technical support)	15 (12.7%)	39 (44.8%)	**54 (26.3%)**

**Table 6:** Analysis of confounding factors.

Confounding Factor	Category	AI Acceptance (%)	Statistical Significance
**Age**	<40 years	**76%**	-
	>60 years	51% (favourable), 20% (against)	-
**Gender**	Male	**68%**	***p* = 0.04** (Chi-square test)
	Female	**60%**	
**Education level**	Higher education	**74%**	-
	Lower education	**58%**	
**Digital literacy**	High digital proficiency	**84%**	***p* < 0.001** (Kruskal–Wallis test)
	Low digital proficiency	**42%**	
**Questionnaire completion method**	Independent (73.7% of total)	Higher AI acceptance	-
	With assistance (26.3% of total)	More sceptical	***p* = 0.03** (Mann–Whitney U test)

**Table 7:** Questionnaire reliability—Cronbach’s Alpha coefficient and test–retest reliability.

Questionnaire Dimension	Number of Items	Cronbach’s Alpha	Test–Retest Reliability (R)	Interpretation
Demographic data	3	-	-	Not applicable
General knowledge of artificial intelligence	2	0.78	0.82	Good consistency
Perceptions of artificial intelligence benefits	5	0.84	0.87	Very good consistency
Challenges and limitations of artificial intelligence	4	0.81	0.83	Good consistency
Acceptance of artificial intelligence in clinical practice	3	0.79	0.80	Good consistency
Data confidentiality and ethics	4	0.85	0.88	Very good consistency
Future use of artificial intelligence in rheumatology	4	0.82	0.86	Very good consistency
General feedback on artificial intelligence	2	0.77	0.81	Good consistency

**Table 8:** Level of acceptance of artificial intelligence in medical practice.

Level of AI Acceptance	Healthcare Professionals (N = 118)	Patients with RA (N = 87)	Total (%)
**Very favourable** (AI is essential and should be fully integrated)	37 (31.4%)	24 (27.6%)	61 (29.8%)
**Favourable** (AI can improve RA management but with limitations)	45 (38.1%)	30 (34.5%)	75 (36.6%)
**Neutral** (No clear opinion on AI in rheumatology)	22 (18.6%)	20 (23.0%)	42 (20.5%)
**Reluctant** (AI raises concerns but could be useful in certain contexts)	10 (8.5%)	9 (10.3%)	19 (9.3%)
**Very reluctant** (AI should not be used in rheumatology)	4 (3.4%)	4 (4.6%)	8 (3.9%)

**Table 9:** Relationship between AI experience and perception of AI utility in RA.

Prior AI Experience	N	Consider AI Useful (%)	Neutral (%)	Reluctant Towards AI (%)
**Had used AI before**	74	84.5%	9.5%	6%
**Had not used AI before**	131	57.3%	23.7%	19%
**Total**	205	66.8%	18.5%	14.7%

**Table 10:** Relationship between AI knowledge and level of acceptance in rheumatology.

AI Knowledge Level	N	Support AI Integration (%)	Neutral (%)	Against AI (%)
**Very good**	43	93.0%	5%	2%
**Good**	82	77.1%	15%	7.9%
**Neutral**	50	60.2%	24%	15.8%
**Low/None**	30	42.1%	33%	24.9%
**Total**	205	66.3%	18.9%	14.8%

**Table 11:** Most common barriers to AI use.

Main Barrier Identified	Healthcare Professionals (N = 118)	Patients (N = 87)	Total (%)
**High costs**	39 (33.1%)	35 (40.2%)	36.1%
**Lack of staff training**	47 (39.8%)	29 (33.3%)	37.6%
**Data privacy concerns**	19 (16.1%)	24 (27.5%)	21.0%
**Limited insurance coverage**	9 (7.6%)	5 (5.7%)	6.8%
**Other reasons**	4 (3.4%)	4 (4.6%)	4.0%

**Table 12:** Differences in attitudes towards AI between healthcare professionals and patients with RA.

Aspect Analysed	Healthcare Professionals (N = 118)	Patients with RA (N = 87)
Consider AI beneficial for RA diagnosis	73%	62%
Concerns about data privacy	19%	27.5%
Acceptance of AI for disease monitoring	65%	49%
Concern about algorithm accuracy	35%	18%
Concern about the impact on the doctor-patient relationship	12%	18%

**Table 13:** Influence of demographic variables on the perception of AI use in rheumatology.

Demographic Variable	Category	AI Acceptance (%)	Main Concerns	*p*-Value
**Age**	<40 years	**76%**	AI integration into existing systems	***p*** ** = 0.01**
	40–60 years	64%	High technology implementation costs	***p*** ** = 0.03**
	>60 years	**51%**	Complexity of AI use, lack of trust	***p*** ** = 0.02**
**Gender**	Male	**68%**	Lack of data standardisation	***p*** ** = 0.04**
	Female	**60%**	Privacy concerns and diagnostic errors	***p*** ** = 0.02**
**Education level**	Higher education	**74%**	Reliability of AI algorithms	***p*** ** = 0.01**
	Secondary education	**58%**	Dependence on technology in medical decisions	***p*** ** = 0.03**

## Data Availability

The database used is from the hospital’s information system database. Considering data protection regulations, we cannot provide access to the original database.

## Supplementary Materials

The following supporting information can be downloaded at: https://www.mdpi.com/article/10.3390/medicina61040689/s1, File S1: Questionnaire: Perception of AI Use in the Diagnosis and Management of RA.
