# Supplementary material for: The Role of Artificial Intelligence in the Diagnosis and Management of Rheumatoid Arthritis
Source: Medicina (Kaunas). 2025 Apr 9;61(4):689. doi: 10.3390/medicina61040689 (PMC12028963; doi:10.3390/medicina61040689)
Supplement: Supplementary file 1 [file medicina-61-00689-s001.zip › medicina-3472254-supplementary.pdf]

File S1. Questionnaire: Perception of AI Use in the Diagnosis and Management of RA

Dear Participant,

Thank you for your willingness to participate in this study exploring the use of Artificial Intelligence (AI) in the diagnosis and management of rheumatoid arthritis.

The aim of this study is to understand physicians' perceptions, experiences, and opinions regarding the integration of AI technology into medical practice. Your responses will contribute to the development of more efficient solutions, better tailored to clinical needs.

The questionnaire is structured to gather relevant information about your professional experience, perceptions of the benefits and limitations of AI, and the potential of this technology to enhance medical practice. Completing the questionnaire takes approximately **10 min**.

Participation is voluntary, and responses will be treated anonymously and used exclusively for scientific purposes. In compliance with data protection regulations, no personally identifiable information will be collected, and all data will be analysed and presented in aggregated form.

By completing this questionnaire, you confirm your agreement to participate in this study. If you have any questions or concerns, please do not hesitate to contact the research team.

Thank you for your valuable contribution and support!

Section 1: Demographic Data

1. What is your role in the healthcare field?

a. Rheumatologist
b. General practitioner
c. Researcher
d. Patient
e. Other (please specify): ___________

2. How many years of experience do you have in your field?

a. <5 years
b. 5–10 years
c. 10–20 years
d. >20 years

3. Do you have prior experience with AI-based technologies?

a. Yes (continue questionnaire)
b. No (end of questionnaire)

Section 2: General Knowledge about AI

4. How well do you understand the concept of Artificial Intelligence (AI)?

a. Very well
b. Well
c. Neutral
d. Slightly
e. Not at all

5. To what extent do you think AI can bring benefits to the diagnosis of rheumatic diseases?

a. To a very large extent
b. To a large extent
c. Neutral
d. To a small extent
e. To a very small extent
f. Not at all

Section 3: Use of AI in Rheumatoid Arthritis

6. What do you consider to be the greatest advantage of using AI in the diagnosis of rheumatoid arthritis? (You may select multiple answers)
a. Early disease diagnosis
b. Reduction of medical errors
c. Big data analysis for identifying clinical patterns
d. Reduced time required for patient evaluation
e. Other (please specify): ___________

7. To what extent do you believe AI could be useful in the management of rheumatoid arthritis?
a. To a very large extent
b. To a large extent
c. Neutral
d. To a small extent
e. To a very small extent
f. Not at all

8. Have you ever used an AI-based tool for diagnosis or treatment?
a. Yes (examples, if possible): ___________
b. No

9. What types of AI technologies do you consider most promising in this field?
a. Machine learning algorithms for blood test interpretation
b. Neural networks for imaging analysis (MRI, X-rays)
c. Recommendation systems for personalised treatments
d. Digital platforms for patient symptom monitoring
e. Other (please specify): ___________

Section 4: Challenges and Limitations
10. What do you consider to be the greatest challenge in using AI for rheumatoid arthritis?
a. High implementation costs
b. Lack of medical staff training
c. Uncertainty regarding diagnostic accuracy
d. Patient data confidentiality concerns
e. Other (please specify): ___________

11. To what extent do you have reservations about using AI in clinical practice?
a. To a very large extent
b. To a large extent
c. Neutral
d. To a small extent
e. To a very small extent
f. Not at all

Section 5: Future Perspectives
12. To what extent do you believe AI should be integrated into routine rheumatology practice?
a. To a very large extent
b. To a large extent
c. Neutral
d. To a small extent
e. To a very small extent
f. Not at all

13. What should be prioritised in AI development for rheumatoid arthritis?
a. Increasing accessibility to technology
b. Improving algorithm accuracy
c. Establishing clear ethical protocols
d. Educating medical staff on AI use
e. Other (please specify): ___________

14. To what extent would you be willing to recommend AI use for your patients?
a. To a very large extent
b. To a large extent
c. Neutral
d. To a small extent
e. To a very small extent
f. Not at all

Section 6: AI Integration in Clinical Practice
15. How do you think AI can support the clinical decision-making process in rheumatoid arthritis?
a. AI can fully guide clinical decisions
b. AI can support physicians, but decisions should be made by them
c. AI is only useful for analysing complex data
d. AI has no relevant role in decision-making

16. At which stage of the medical process do you believe AI would have the greatest impact? (Select all relevant answers)
a. Early diagnosis
b. Monitoring disease progression
c. Prescribing personalised treatments
d. Preventing complications
e. Other (please specify): ___________

Section 7: Patient Acceptance of AI Technologies
17. Do you think patients would accept a diagnosis provided by an AI algorithm without physician intervention?
a. Yes, without reservations
b. Yes, but only with validation from a physician
c. No, patients would prefer human interaction
d. Not sure

18. What would improve patient trust in AI use for rheumatoid arthritis? (You may select multiple answers)
a. Educating patients about how AI works
b. Rigorous clinical validation of AI tools
c. Physician involvement in the diagnostic process
d. Transparency in the use of personal data
e. Other (please specify): ___________

Section 8: Data Privacy and Ethics
19. How concerned are you about the use of personal health data in AI algorithms?
a. Very concerned
b. Fairly concerned
c. Neutral
d. Slightly concerned

e. Not concerned at all

20. What do you consider the greatest risk regarding data confidentiality in AI use?
a. Unauthorised data access
b. Commercial use of data without consent
c. Errors in data anonymisation
d. Other (please specify): ___________

Section 9: Future of AI in Rheumatology
21. What other AI applications could be developed for rheumatoid arthritis patients?
a. Patient education platforms
b. Predictors of treatment response
c. Virtual assistants for daily symptom tracking
d. Wearable devices monitoring disease activity
e. Other (please specify): ___________

22. In what timeframe do you believe AI will become standard in rheumatoid arthritis care?
a. Within the next 1–3 years
b. Within the next 5–10 years
c. In more than 10 years
d. I don't believe it will become standard

Thank you!
